# Simultaneous Determination of Paracetamol, Ibuprofen, and Caffeine in Tablets by Molecular Absorption Spectroscopy Combined with Classical Least Square Method

**DOI:** 10.3390/molecules27092657

**Published:** 2022-04-20

**Authors:** Binh Thuc Tran, Tuyen Ngoc Tran, Ai My Thi Tran, Giang Chau Dang Nguyen, Quynh Trang Thi Nguyen

**Affiliations:** 1 Chemistry Department, University of Sciences, Hue University, Hue 530000, Vietnam; tntuyen@hueuni.edu.vn (T.N.T.); aimy.chem@hueuni.edu.vn (A.M.T.T.); chaundg@hueuni.edu.vn (G.C.D.N.); 2 Faculty of Environmental Sciences, Saigon University, Ho Chi Minh City 700000, Vietnam; trangntm@sgu.edu.vn

**Keywords:** paracetamol, ibuprofen, caffeine, classical least-square, simultaneous, spectroscopy

## Abstract

In this paper, the classical least-squares (CLS) method with molecular absorption spectrophotometric measurement was used to determine simultaneously paracetamol (PAR), ibuprofen (IBU), and caffeine (CAF) in tablets. The absorbance spectra of the standard solutions and samples were measured over a wavelength from 220 to 300 nm with a 0.5 nm step. The concentration of PAR, IBU, and CAF in the sample solutions was calculated by using Visual Basic for Applications (VBA) and a program called CLS-Excel written in Microsoft Excel 2016. The method and the CLS-Excel program were tested on mixed standard laboratory samples with different PAR, IBU, and CAF concentration ratios, and they showed only small errors and a satisfying repeatability. An analytical procedure for tablets containing PAR, IBU, and CAF was developed. The reliability of the procedure was proved via the recovery and repeatability of the analysis results with an actual tablet sample and by comparing the mean contents of active substances in the tablets obtained from the analytical procedure with the HPLC method. The procedure is simple with a reduced cost compared with the HPLC standard method.

## 1. Introduction

Paracetamol (PAR), ibuprofen (IBU), and caffeine (CAF) are the main active ingredients widely used in multicomponent pharmaceuticals. PAR is a common pain reliever and fever reducer. IBU is a nonsteroidal anti-inflammatory drug with good analgesic and antipyretic effects. CAF is a methylated xanthine that stimulates the central nervous system, reduces feelings of fatigue and drowsiness, and increases brain excitement and sensory perception, thereby helping humans to work more effectively. The combination of these ingredients in tablets enhances the healing effect [1].

Quality control of multicomponent pharmaceutical products requires fast and reliable analytical techniques. The UV-Vis spectroscopy method is commonly used in laboratories due to its simplicity and low equipment cost. However, the quantitative analysis of pharmaceutical products containing many components having overlap spectra is often difficult. To analyze them by conventional UV-Vis method, we must often extract specific substance or mask substances which interfere with the analytical procedure. Thus, the procedure becomes complicated, consuming much time, chemicals, and solvent, with poor reliability. Currently, numerous UV-Vis spectroscopy methods combined with chemometrics have been developed to analyze simultaneously substances with overlapping absorption spectra. These methods often use entire spectrum data and computer programs to calculate, eliminate measurement errors, and statistically assess a large amount of data to give reliable and useful information. In particular, they allow us to calculate the concentrations of substances in multicomponent solutions with high accuracy without separation or masking. This advantage enables researchers to design simple, low-cost, short analytical procedures with high reliability. The UV-Vis molecular absorption spectrophotometric method is coupled with chemometrics used for simultaneous determination of substances in multicomponent pharmaceuticals. Such chemometrics include the classical least-squares (CLS) [1,2], partial least-squares (PLS) [2,3,4], principal component regression (PCR) [2,4], artificial neural network (ANN) [1,4], derivative [5,6,7], and Kalman filter [8] methods, as well as others. As we know, the absorption spectra of PAR, IBU, and CAF overlap to a great extent in the ultraviolet region. Many methods have been developed for the determination of PAR, IBU, and CAF in multicomponent drugs, including standard methods [9,10], spectroscopy [3,5,6,7], and chromatography [11]. To the best of our knowledge, there is no published paper concerning the use of the CLS method with full spectrum to simultaneously determine PAR, IBU, and CAF in drugs. Thus, in this paper we apply the CLS method for the full spectrum to simultaneously determine PAR, IBU, and CAF in drugs using Visual Basic for Applications (VBA) and a self-written program called CLS-Excel written on Microsoft Excel 2016.

## 2. Apparatus, Materials and Methods

### 2.1. Apparatus and Chemicals

#### 2.1.1. Apparatus

A Cary 60 UV-Vis spectrophotometer (Agilent, Santa Clara, CA, USA) was in a wavelength range of 190–990 nm, connected to a computer with Cary WinUV software for storing spectral data as an excel spreadsheet. Other basic laboratory equipment was also used.

#### 2.1.2. Chemicals

Paracetamol 99.9%, ibuprofen 100.1%, and caffeine 99.9% conforming with Vietnamese pharmaceutical standards were supplied from the Central Institute for Drug Testing, Vietnam.

A drug sample of Ibuparavic, containing paracetamol (300 mg/tablet), ibuprofen (200 mg/tablet), and caffeine (20 mg/tablet), was purchased from Thanh Nam Pharmaceutical Manufacturing and Trading Co., Ltd., Ho Chi Minh City, Vietnam. The production batch number was 601119, and the sample was produced on 11 January 2019 and expired on 10 January 2022. A box has 10 blisters with 10 hard capsules each, and the registration number is GC 318-19.

Distilled water and methanol (Merck) were also used.

##### Paracetamol, Ibuprofen, and Caffeine Standard Solutions

First, stock solutions with a 50 µg/mL concentration were prepared as follows: precisely 12.5 mg of each preparation was placed in a 250 mL volumetric flask with methanol, appropriately shaken, and made up to the mark. Then 50 mL of each solution was transferred to a 100 mL volumetric flask and made up to the mark with methanol to obtain a 25 µg/mL working solution. Finally, 10 mL of the working solution was placed in a 25 mL volumetric flask and made up to the mark with methanol to a 10 µg/mL PAR and IBU standard solution. For preparing a 5 µg/mL CAF standard solution, 5 mL of the working solution was used.

##### Mixed Experimental Solutions 

The working solution of PAR, IBU, and CAF was mixed with different volume ratios. The standard and working solutions were used to verify the reliability of the method.

### 2.2. Analytical Procedure

The theoretical basis of the classical least-squares method is as follows:

For multicomponent systems, the absorbance is cumulative. We use Beer’s law for a system of *n* components and *m* wavelengths (*m* > *n*). Let *e_i_* = ε*_i_* × *b*, *Y_i_* = *A_i_*, and *x_i_* = *C_i_*, where ε*_i_*is the molecular absorptivity of the *i*-th component; *C_i_* is the concentration of the *i*th component in the mixture; and *A_i_* is the absorbance of the mixed solution measured at the *i*th wavelength. A system of linear equations is obtained with *m* equations and *n* unknowns as follows:

(1) $${\left\{ \begin{array}{l} Y_{1}=e_{11}x_{1}+e_{12}x_{2}+\ldots +e_{1i}x_{i}+\ldots +e_{1n}x_{n}\\ Y_{2}=e_{21}x_{1}+e_{22}x_{2}+\ldots +e_{2i}x_{i}+\ldots +e_{2n}x\\ \vdots \\ Y_{j}=e_{j}{}_{1}x_{1}+e_{j}{}_{2}x_{2}+\ldots +e_{ji}x_{i}+\ldots +e_{jn}x_{n}\\ \vdots \\ Y_{m}=e_{m}{}_{1}x_{1}+e_{m}{}_{2}x_{2}+\ldots +e_{m}{}_{i}x_{i}+\ldots +e_{mn}x_{n} \end{array}\right.}_{\;}$$

The molecular absorbance measured at the *j*th wavelength is *y_j_*. This parameter is often erroneous, and it is different from the actual value *Y_j_* by a value *s_j_*, where *s_j_* is the measurement residual:

*s_j_* = *y_j_* − *Y_j_* (2)

The function representing the total squared error *S* is

(3) $$S=\overset{m}{\underset{j=1}{\sum }}{\left(y_{j}-Y_{j}\right)}^{2}=\overset{m}{\underset{j=1}{\sum }}{\left[y_{j}-\left(e_{j1}x_{1}+e_{j2}x_{2}+\ldots e_{ji}x_{i}+\ldots +e_{jn}x_{n}\right)\right]}^{2}$$

For *S* to be minimized, the derivative of *S* with respect to *x_i_* must be 0. If we take the derivative of *S* with respect to *x*_1_ and let the derivative equal 0, we get the following equation:

$$\frac{dS}{dx_{1}}=2\overset{m}{\underset{j=1}{\sum }}\left[y_{j}-\left(e_{j1}x_{1}+e_{j2}x_{2}+\ldots e_{ji}x_{i}+\ldots +e_{jn}x_{n}\right)\right].\left(-e_{j1}\right)=0$$

Transforming this equation, we get

(4) $$\overset{m}{\underset{j=1}{\sum }}e_{j1}^{2}x_{1}+\overset{m}{\underset{j=1}{\sum }}e_{j1}e_{j2}x_{2}+\ldots +\overset{m}{\underset{j=1}{\sum }}e_{j1}e_{ji}x_{i}+\ldots +\overset{m}{\underset{j=1}{\sum }}e_{j1}e_{jn}x_{n}-\overset{m}{\underset{j=1}{\sum }}e_{j1}y_{j}=0_{\;}$$

Similarly, we also take the derivative *S* with respect to the remaining *x_i_* and let these derivatives equal 0. Combining this equation with Equation (4), we get the following system of equations:

(5) $$\left\{ \begin{array}{l} x_{1}\overset{m}{\underset{j=1}{\sum }}e_{j1}^{2}+x_{2}\overset{m}{\underset{j=1}{\sum }}e_{j1}e_{j2}+\ldots +x_{i}\overset{m}{\underset{j=1}{\sum }}e_{j1}e_{ji}+\ldots +x_{n}\overset{m}{\underset{j=1}{\sum }}e_{j1}e_{jn}-\overset{m}{\underset{j=1}{\sum }}e_{j1}y_{j}=0\\ x_{1}\overset{m}{\underset{j=1}{\sum }}e_{j1}e_{j2}+x_{2}\overset{m}{\underset{j=1}{\sum }}e_{j2}^{2}+\ldots +x_{i}\overset{m}{\underset{j=1}{\sum }}e_{j2}e_{ji}+\ldots +x_{n}\overset{m}{\underset{j=1}{\sum }}e_{j2}e_{jn}-\overset{m}{\underset{j=1}{\sum }}e_{j2}y_{j}=0\\ x_{1}\overset{m}{\underset{j=1}{\sum }}e_{j1}e_{ji}+x_{2}\overset{m}{\underset{j=1}{\sum }}e_{j2}e_{ji}+\ldots +x_{i}\overset{m}{\underset{j=1}{\sum }}e_{ji}^{2}+\ldots +x_{n}\overset{m}{\underset{j=1}{\sum }}e_{ji}e_{jn}-\overset{m}{\underset{j=1}{\sum }}e_{ji}y_{j}=0\\ x_{1}\overset{m}{\underset{j=1}{\sum }}e_{j1}e_{jn}+x_{2}\overset{m}{\underset{j=1}{\sum }}e_{j2}e_{jn}+\ldots +x_{i}\overset{m}{\underset{j=1}{\sum }}e_{ji}e_{jn}+\ldots +x_{n}\overset{m}{\underset{j=1}{\sum }}e_{jn}^{2}-\overset{m}{\underset{j=1}{\sum }}e_{jn}y_{j}=0_{\;} \end{array}\right.$$

Let

(6) $$a_{ki}=\overset{m}{\underset{j=1}{\sum }}e_{ji}e_{jk};\;b_{k}=\overset{m}{\underset{j=1}{\sum }}e_{jk}y_{j}$$

where $i=\bar{1\ldots n}$; $k=\bar{1\ldots n}$.

The system of equations can be summarized as follows:

(7) $${\left\{ \begin{array}{l} a_{11}.x_{1}+a_{12}.x_{2}+\ldots +a_{1i}x_{i}+\ldots +a_{1n}.x_{n}=b_{1}\\ a_{21}.x_{1}+a_{22}.x_{2}+\ldots +a_{2i}x_{i}+\ldots +a_{2n}.x_{n}=b_{2}\\ \vdots \\ a_{k}{}_{1}.x_{1}+a_{k}{}_{2}.x_{2}+\ldots +a_{ki}x_{i}+\ldots +a_{kn}.x_{n}{}_{}=b_{k}\\ \vdots \\ a_{n}{}_{1}.x_{1}+a_{n}{}_{2}.x_{2}+\ldots +a_{ni}x_{i}+\ldots +a_{nn}.x_{n}=b_{n} \end{array}\right.}_{\;}$$

The values of *a_ki_* and *b_k_* in the system of Equation (7) are calculated from the initial experimental values of *e_ji_* by using Equation (6). The system of Equation (7) is a system of linear equations consisting of *n* equations with *n* unknowns. Solving this system of equations with the Gaussian reduction method, we have the concentration of the components *x_i_*. The concentration of the components in the sample solution was calculated by using the CLS-Excel program.

The advantage of this method is that it uses all spectral data to create a system of linear equations with more equations than unknowns. Then, by transforming this system of equations with the least-squares technique, we obtain a system with an equal number of equations and unknowns. As a result, the error becomes minimal, thus enhancing accuracy. The concentration of the substances in the sample solution is determined rapidly thanks to the program. The method can be applied to the substances in the mixtures with the components’ complex absorption spectra overlapping.

The steps for measuring and calculating the concentration of substances are as follows:

- Preparing standard solutions of each component to be determined and the sample solutions containing their mixtures.
- Scanning the spectrum of the solutions at an appropriate wavelength range to obtain CSV files in the form of an excel spreadsheet.
- Running the CLS-Excel program for the data from the excel files to calculate the concentration of components in the mixed solution and their relative error.

### 2.3. Statistical Parameters

#### 2.3.1. Relative Error

The relative error between the determined concentration and the preparation concentration (RE%) was calculated according to Equation (8)

(8) $$RE\left(\%\right)=\frac{\left(C-C_{0}\right).100}{C_{0}}$$

where *C* is the determined concentration (µg/mL) and *C*_0_ is the concentration of the known standard solution (µg/mL).

#### 2.3.2. Repeatability

Repeatability was assessed by using the relative standard deviation value (RSD%):

(9) $$RSD\left(\%\right)=\frac{S.100}{C_{mean}}$$

where *S* is the standard deviation and *C_mean_* is the mean concentration after *n* measurements (µg/mL). For in-laboratory quality control, method repeatability is satisfactory when the RSD% values obtained are less than 1/2RSD_Horwitz_ [12,13]

RSD_Horwitz_ = 2^(1−0.5×lg*C*)^ (10)

where *C* is the concentration expressed as a power (for example, *C* = 5 µg/mL = 5 × 10^–6^).

#### 2.3.3. Accuracy

a. Recovery

The recovery of the method was calculated based on the standard addition according to Equation (11)

(11) $$Rev\left(\%\right)=\frac{\left(C_{2}-C_{1}\right).100}{C_{\mathrm{add}}}$$

where *C*_2_ (µg/mL) is the determined concentration of the sample solution after standard addition; *C*_1_ (µg/mL) is the determined concentration of the sample solution before standard addition; and *C*_add_ (µg/mL) is the standard addition concentration [14].

b. Comparison of the proposed method with the HPLC standard method

The basic information of the HPLC standard method [10] to analyze the tablet containing PAR, IBU, and CAF is as follows:

First: Determination of IBU only: Stationary phase: C18 (100 × 4.6 mm; 5 µm); Mobile phase: Acid phosphoric 0.01 M: Acetonitril 60:40 (V:V); Detector: Diode Array, UV at λ = 224 nm; Flowrate: 1.0 mL/min;

Second: simultaneous determination of PAR and CAF: Stationary phase: C18 (100 × 4.6 mm; 5 µm); Mobile phase: Water-methanol-glacial acid acetic (69:28:3) (V); Detector: Diode Array at λ = 275 nm; Flowrate: 2.0 mL/min.

According to [15], to determine the method’s accuracy, we analyze the same sample repeatedly with the proposed method and the standard method. Then we compare the two sample mean values by using the Student’s *t*-test.

(12) $$t_{\exp}=\frac{|{\bar{X}}_{A}-{\bar{X}}_{B}|}{\sqrt{\left(S_{A}^{2}/n_{A}+S_{B}^{2}/n_{B}\right)}}$$

where *t*_exp_ is the experimental student value; ${\bar{X}}_{A}$ and ${\bar{X}}_{B}$ are the mean value of methods *A* and *B*; *n_A_* and *n_B_* are the number of repeat measurements of methods *A* and *B*; and $s_{A}^{2}$, $s_{B}^{2}$ are the variance of the two methods.

Finally, we compare the *t*_exp_ value with the theoretical student value *t*(α, ν), where α is the significance level (usually taken as 0.05) and ν is the degrees of freedom determined above. If *t*_exp_ < *t*(α,ν), the mean values of the two methods are not significantly different.

### 2.4. Actual Sample Treatment and Calculation of the Content of Substances

#### 2.4.1. Sample Treatment

Twenty tablets from the same production batch were weighed, and the average weight was determined (*M*). Then the tablets were ground to fine powder in an agate mortar. An amount of powder equal to 0.7 to 1.0 of the average tablet weight was placed into a 250 mL beaker containing 150 mL of methanol. The content of the beaker was sonicated for 30 min and quantitatively transferred to a 250 mL volumetric flask, made up to the mark with methanol and thoroughly mixed. The solution was then filtered through blue-band filter paper; the first 10 mL of the filtrate was discarded. Next, 10 mL of the filtrate was transferred to a 100 mL volumetric flask, made up to the mark with methanol, and thoroughly mixed (solution 1). Again, 10 mL of solution 1 was diluted to 100 mL with methanol to obtain solution 2. Finally, solution 2 was subjected to UV-Vis absorption determination, and the CLS-Excel program (please see the Supplementary Materials) was used to calculate the concentration of the active substances. The concentration of the active ingredients from another 20 pills from the same batch was determined simultaneously with the HPLC method.

#### 2.4.2. Calculation of the Content of Substances

The content of active ingredients in one tablet was determined from the formula (13)

*H* (mg/tablet) = *C_m_* × 100 × (100/10) × (250/10) × (1/1000) × (*M*/*m*) = 25 × *C_m_* × (*M*/*m*) (13)

where *C_m_* (µg/mL) is the concentration of each active ingredient determined in the sample solution; *m* is the weight of the sample (mg); and *M* is the average tablet weight (mg).

## 3. Results and Discussion

### 3.1. Accuracy and Repeatability of the Analytical Method on Laboratory Samples

**Figure 1:**
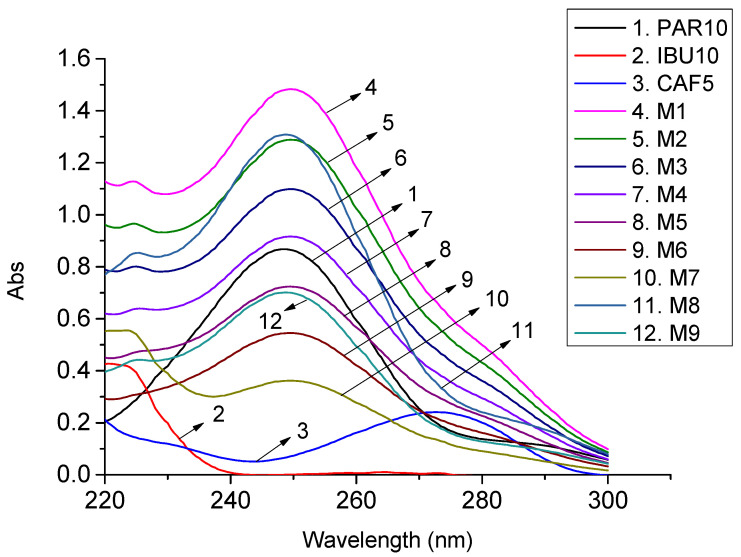
UV absorption spectra of standard solutions and laboratory mixture solutions with different concentration ratios (Standard solution (µg/mL): PAR 10, IBU 10, CAF 5; PAR/IBU/CAF mixed solution (µg/mL): M1 (16:12:7); M2 (14:10:6); M3 (12:8:5); M4 (10:6:4); M5 (8:4:3); M6 (6:2:2); M7 (4:10:1); M8 (15:10:1; M9 (8:5:0.5)).

From the working standards of PAR 25 μg/mL, IBU 25 μg/mL, and CAF 25 μg/mL, prepare individually 10 µg/mL PAR and IBU standard solutions and 5 µg/mL CAF as described in Section 2.1.2 and their mixture solutions at different concentration ratios (Table 1). The standard solutions and the mixture solutions were measured three times. The solutions were spectroscopically scanned in the range of 220–300 nm with 0.5 nm intervals. The relative error between the determined concentration and the preparation concentration of PAR, IBU, and CAF in the mixed solutions was calculated according to the CLS-Excel program, and the corresponding relative standard deviation (RSD%) of the analytical results was also calculated. The absorption spectra of the standard solutions and laboratory mixture solutions are illustrated in Figure 1. The concentrations of PAR, IBU, and CAF in the mixtures and statistical data are presented in Table 1.

Table 1 shows that at different concentration ratios the errors of the concentrations of PAR, IBU, and CAF determined with the CLS method are from −1.40 to 1.12% and that the RSD% values are also small (RSD%_max_ = 1.103) and less than 1/2RSD_Horwitz_. Therefore, the method’s accuracy and repeatability are satisfactory for mixed laboratory solutions with different concentration ratios.

### 3.2. Simultaneous Quantification of PAR, IBU, and CAF in Drug Samples

The characteristics of ibuparavic tablets were described in Section 2.1.2 with the average tablet weight of 0.5265 g.

The samples were treated as described in Section 2.4.1 with precisely 526.5 mg of powder. The entire spectrum of the sample solution was scanned in the wavelength range of 220–300 nm, with a 0.5 nm step. The concentration of PAR, IBU, and CAF in the sample solutions was determined with the CLS-Excel program, and their content was calculated from the Formula (13).

**Figure 2:**
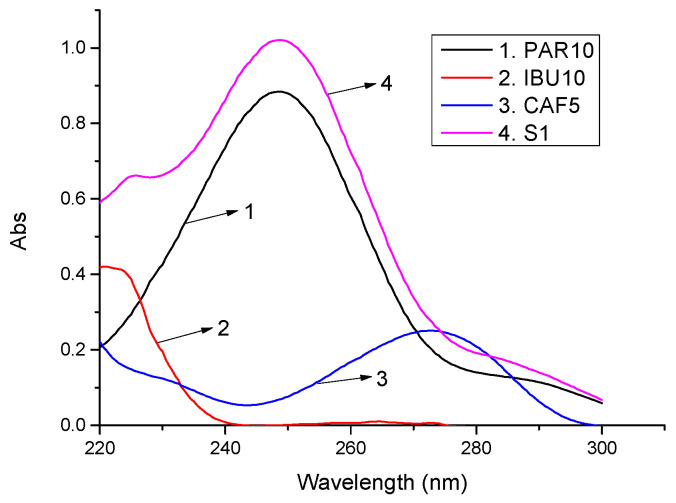
Absorption spectra of standard solutions and sample solutions of ibuparavic (standard solution 10 µg/mL: PAR (1), IBU (2); standard solution 5 µg/mL: CAF (3); S1: Ibuparavic drug sample solution (4)).

The absorption spectra of the standard solutions and sample solutions of ibuparavic are presented in Figure 2, and the content of the active ingredient is displayed in Table 2.

The data show that the method is highly reproducible with all three components (RSD% < 1.2). The content of each substance in the ibuparavic tablets is as follows: PAR: 286.95 ± 1.37 mg, IBU: 194.50 ± 2.49 mg, and CAF 20.08 ± 0.52 mg. This content is consistent with that reported on the label of these tablets and also agrees with the quality standards required by Vietnam’s Ministry of Health: PAR 300 mg ± 5% (285–315 mg), IBU 200 mg ± 5% (190–210 mg), and CAF 20 mg ± 5% (19–21 mg).

### 3.3. Accuracy Verification

#### 3.3.1. Recovery

**Figure 3:**
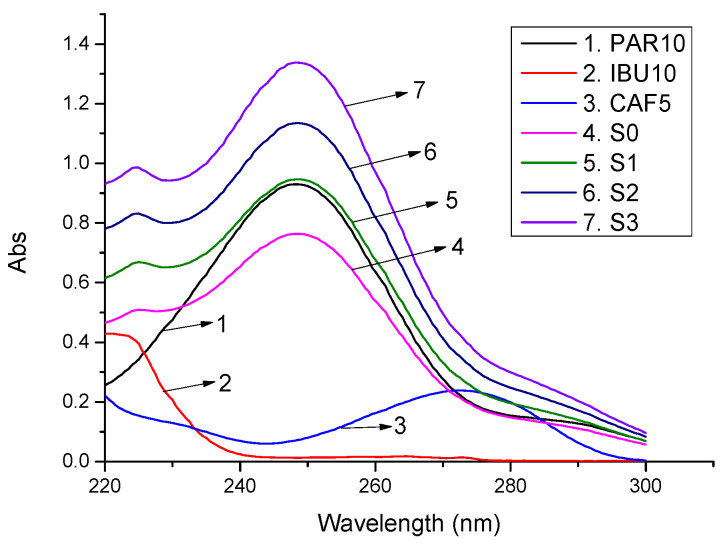
Absorbance spectra of standard solutions, sample solution, and standard addition solutions (standard solution (µg/mL): PAR 10, IBU 10, CAF 5; S0: drug sample without standard S0 (*m* = 0.7 *M*_tablets_); PAR/IBU/CAF mixed solution (µg/mL): S1 (2:2:0.5); S2 (4:4:1); S3 (6:6:1.5)).

Four batches of the sample powder equal to 0.7 times the average tablet weight were weighed. No standard addition was carried out for the first batch. The remaining three batches were added with PAR, IBU, and CAF with increasing amounts of standard. The samples were treated as described in Section 2.4.1. Measurements were carried out for the spectra of standard solutions PAR 10 µg/mL, IBU 10 µg/mL, and CAF 5 µg/mL, the sample solution without standard (S0), and sample solutions after adding standards (S1, S2, S3). The concentration of PAR, IBU, and CAF in the standard and sample solutions was calculated with the CLS-Excel program. The spectra of the standard solutions and the sample solutions are shown in Figure 3. The concentration of the standard additions and that of the sample without and with the added standard is presented in Table 3. The recovery of the CLS-Excel method was calculated from Equation (11).

Table 3 shows that the method’s recovery is satisfactory: 92.80–98.30% for PAR, 95.73–104.05% for IBU, and 94.60–101.80% for CAF. All recovery values are within the allowable range required by AOAC [13].

#### 3.3.2. Comparison of CSL-Excel and HPLC Methods

To objectively evaluate the accuracy of our method, we compared the content of the active ingredients in Ibuparavic tablets with those determined with the standard HPLC method performed by the Centre for Drug, Food, and Cosmetic Testing in Thua Thien Hue, Vietnam [10]. The comparison was carried out statistically [15] (Table 4).

The results in Table 4 show that the calculated t-values are smaller than the t-theory values, indicating that the CLS and HPLC methods are statistically identical at α = 0.05. Thus, we can say that the method has a satisfying accuracy.

## 4. Conclusions

An analytical procedure for simultaneous determination of PAR, IBU, and CAF in tablets was developed by using the molecular absorption spectrophotometric method with the entire spectrum, coupled with the classical least-squares technique. The concentration of PAR, IBU, and CAF in the sample solutions was calculated by using Visual Basic for Applications (VBA) and a self-made program called CLS-Excel written in Microsoft Excel 2016. The analytical procedure has satisfactory repeatability with an RSD% less than or equal to 1.052. The recoveries obtained for PAR, IBU, and CAF ranged from 92.80 to 98.30, 95.73 to 104.05, and 94.60 to 101.80%, respectively. The content of PAR, IBU, and CAF in the drug sample Ibuparavic analyzed with the procedure is consistent with that of the HPLC method at the 0.05 significance level.

## Figures and Tables

**Table 1:** Concentration of PAR, IBU, and CAF in the mixture, and their RE and statistics.

Sample	Conc. Ratio (μg/mL) PAR/IBU/CAF	Run Order	PAR			IBU			CAF		
			*C*_PAR_ (μg/mL)	RE (%)	Statistics	*C*_IBU_ (μg/mL)	RE (%)	Statistics	*C*_CAF_ (μg/mL)	RE (%)	Statistics
M1	16:12:7	1 2 3	16.091 16.179 16.161	0.57 1.12 0.82	*C*_mean_ = 16.144 RSD (%) = 0.288 1/2RSD_H_ = 5.270 RE_mean_ (%) = 0.84	11.858 11.892 11.885	−1.18 −0.93 −0.96	*C*_mean_ = 11.878 RSD (%) = 0.151 1/2RSD_H_ = 5.504 RE_mean_ (%) = −1.02	6.989 6.989 7.000	−0.16 −0.16 0.00	*C*_mean_ = 6.993 RSD (%) = 0.091 1/2RSD_H_ = 5.969 RE_mean_ (%) = −0.11
M2	14:10:6	1 2 3	13.992 14.059 14.013	−0.06 0.42 0.09	*C*_mean_ = 14.021 RSD (%) = 0.244 1/2RSD_H_ = 5.378 RE_mean_ (%) = 0.15	10.004 9.981 10.002	0.04 −0.19 0.02	*C*_mean_ = 9.996 RSD (%) = 0.127 1/2RSD_H_ = 5.657 RE_mean_ (%) = −0.07	5.991 5.980 5.990	−0.15 −0.33 −0.17	*C*_mean_ = 5.987 RSD (%) = 0.102 1/2RSD_H_ = 6.109 RE_mean_ (%) = −0.22
M3	12:8:5	1 2 3	11.943 12.008 11.964	−0.47 0.07 −0.30	*C*_mean_ = 11.992 RSD (%) = 0.277 1/2RSD_H_ = 5.504 RE_mean_ (%) = −0.23	8.023 7.996 7.989	0.29 −0.05 −0.14	*C*_mean_ = 8.003 RSD (%) = 0.224 1/2RSD_H_ = 5.850 RE_mean_ (%) = 0.03	4.957 4.958 4.974	−0.86 −0.84 −0.52	*C*_mean_ = 4.963 RSD (%)= 0.192 1/2RSD_H_ = 6.279 RE_mean_ (%) = −0.71
M4	10:6:4	1 2 3	9.979 10.035 10.004	−0.21 0.35 0.04	*C*_mean_ = 10.006 RSD (%) = 0.280 1/2RSD_H_ = 5.657 RE_mean_ (%) = 0.06	6.040 6.052 6.044	0.67 0.87 0.73	*C*_mean_ = 6.045 RSD (%) = 0.101 1/2RSD_H_ = 6.109 RE_mean_ (%) = 0.76	3.961 3.959 3.969	−0.98 −1.03 −0.78	*C*_mean_ = 3.963 RSD (%) = 0.134 1/2RSD_H_ = 6.493 RE_mean_ (%) = −0.91
M5	8:4:3	1 2 3	7.899 7.937 7.909	−1.26 −0.79 −1.14	*C*_mean_ = 7.918 RSD (%) = 0.247 1/2RSD_H_ = 5.850 RE_mean_ (%) = −1.02	4.023 4.024 4.014	0.58 0.60 0.35	*C*_mean_ = 4.020 RSD (%) = 0.129 1/2RSD_H_ = 6.493 RE_mean_ (%) = 0.50	2.971 2.967 2.971	−0.97 −1.10 −0.97	*C*_mean_ = 2.970 RSD (%) = 0.089 1/2RSD_H_ = 6.781 RE_mean_ (%) = −0.93
M6	6:2:2	1 2 3	5.973 6.027 6.007	−0.45 0.45 0.12	*C*_mean_ = 6.002 RSD (%)= 0.455 1/2RSD_H_ = 6.109 RE_mean_ (%) = 0.04	1.998 2.002 1.992	−0.10 0.10 −0.40	*C*_mean_ = 1.997 RSD (%) = 0.252 1/2RSD_H_ = 7.207 RE_mean_ (%) = −0.13	2.003 1.999 2.003	0.15 −0.05 0.15	*C*_mean_ = 2.002 RSD (%) = 0.115 1/2RSD_H_ = 7.207 RE_mean_ (%) = 0.08
M7	4:10:1	1 2 3	4.009 4.012 4.010	0.23 0.30 0.25	*C*_mean_ = 4.010 RSD (%)= 0.038 1/2RSD_H_ = 6.493 RE_mean_ (%) = 0.26	10.053 9.989 10.035	0.53 −0.11 0.35	*C*_mean_ = 10.026 RSD (%) = 0.329 1/2RSD_H_ = 5.657 RE_mean_ (%) = 0.26	1.006 0.997 1.005	0.60 −0.30 0.50	*C*_mean_ = 1.003 RSD (%) = 0.492 1/2RSD_H_ = 8.000 RE_mean_ (%) = 0.27
M8	15:10:1	1 2 3	14.929 15.060 14.876	−0.47 0.40 −0.83	*C*_mean_ = 14.955 RSD (%)= 0.633 1/2RSD_H_ = 5.322 RE_mean_ (%) = −0.30	9.984 10.010 9.915	−0.16 0.10 −0.85	*C*_mean_ = 9.970 RSD (%) = 0.492 1/2RSD_H_ = 5.657 RE_mean_ (%) = 0.10	1.008 0.997 0.986	0.80 −0.30 −1.40	*C*_mean_ = 0.997 RSD (%) = 1.103 1/2RSD_H_ = 8.000 RE_mean_ (%) = −0.30
M9	8:5:0.5	1 2 3	8.004 7.981 8.044	0.05 −0.24 0.55	*C*_mean_ = 8.010 RSD (%)= 0.398 1/2RSD_H_ = 5.849 RE_mean_ (%) = 0.12	4.974 5.023 4.981	−0.52 0.46 −0.38	*C*_mean_ = 4.993 RSD (%) = 0.531 1/2RSD_H_ = 6.280 RE_mean_ (%) = −0.15	0.507 0.497 0.498	1.40 −0.60 −0.40	*C*_mean_ = 0.501 RSD (%) = 1.099 1/2RSD_H_ = 8.877 RE_mean_ (%) = 0.133

Note: The number of decimal places is taken to represent the calculation result.

**Table 2:** Concentration of PAR, IBU, and CAF in sample solutions and their drug content in Ibuparavic tablets.

Sample	PAR		IBU		CAF	
	*C*_PAR_ (µg/mL)	Content (mg/Tablet)	*C*_IBU_ (µg/mL)	Content (mg/Tablet)	*C*_CAF_ (µg/mL)	Content (mg/Tablet)
S1	11.467	286.68	7.765	194.13	0.812	20.30
S2	11.503	287.58	7.749	193.73	0.795	19.88
S3	11.463	286.58	7.825	195.63	0.802	20.05
Mean	11.478	286.95	7.780	194.50	0.803	20.08
RSD%	0.192		0.515		1.052	
1/2RSD_H_	5.541		5.875		8.269	
%H*	95.65		97.25		100.40	

Note: %H*: % active ingredient compared with labelled content.

**Table 3:** Recovery of CLS-Excel method applied for analyzing Ibuparavic tablets.

Repeated Sample	PAR			IBU			CAF		
	*C*_added_ (µg/mL)	*C*_measured_ (µg/mL)	Rev (%)	*C*_added_ (µg/mL)	*C*_measured_ (µg/mL)	Rev (%)	*C*_added_ (µg/mL)	*C*_measured_ (µg/mL)	Rev (%)
S01	0	8.045		0	5.462	–	0	0.562	
S02		8.000	–		5.502			0.568	–
S03		7.987			5.483			0.564	
Statistics	*C*_measured (mean)_ = 8.011			*C*_measured (mean)_ = 5.482			*C*_measured (mean)_ = 0.565		
	RSD% = 0.380			RSD% = 0.360			RSD% = 0.540		
S11	2.000	9.901	92.80	2.000	7.475	100.65	0.500	1.035	94.60
S12		9.916	95.80		7.543	102.05		1.057	97.80
S13		9.944	98.30		7.564	104.05		1.064	100.00
Statistics	*C*_measured (mean)_ = 9.920			*C*_measured (mean)_ = 7.527			*C*_measured (mean)_ = 1.052		
	RSD% = 0.22			RSD% = 0.61			RSD% = 1.44		
	Rev_mean_ (%) = 95.63			Rev_mean_ (%) = 102.25			Rev_mean_ (%) = 97.47		
S21	4.000	11.824	94.48	4.000	9.551	102.23	1.000	1.578	101.60
S22		11.713	92.83		9.538	100.90		1.585	101.70
S23		11.711	93.10		9.536	101.33		1.582	101.80
Statistics	*C*_measured (mean)_ = 11.749			*C*_measured (mean)_ = 9.542			*C*_measured (mean)_ = 1.582		
	RSD% = 0.55			RSD% = 0.09			RSD% = 0.22		
	Rev_mean_ (%) = 93.47			Rev_mean_ (%) = 101.48			Rev_mean_ (%) = 101.70		
S31	6.000	13.893	97.46	6.000	11.247	96.42	1.500	2.074	100.80
S32		13.777	96.28		11.246	95.73		2.093	101.67
S33		13.774	96.45		11.238	95.92		2.093	101.80
Statistics	*C*_measured (mean)_ = 13.815			*C*_measured (mean)_ = 11.238			*C*_measured (mean)_ = 2.087		
	RSD% = 0.49			RSD% = 0.04			RSD% = 0.52		
	Rev_mean_ (%) = 96.73			Rev_mean_ (%) = 96.02			Rev_mean_ (%) = 101.42		

Note: The number of decimal places is taken to represent the calculation result.

**Table 4:** Comparison of CLS and HPLC methods.

N_0_	Content (H, mg/Tablet)					
	PAR		IBU		CAF	
	CLS	HPLC	CLS	HPLC	CLS	HPLC
1	286.68	287.98	194.13	193.02	20.30	20.37
2	287.58	285.26	193.73	195.90	19.88	20.19
3	286.58	289.18	195.63	195.69	20.05	19.98
*H_mean_*	286.95	287.47	194.50	194.87	20.08	20.18
RSD (%)	0.19	0.73	0.51	0.83	1.05	0.99
*t_cal_*	0.438		0.342		0.622	
*t_theory_* (0.05;4)	2.78		2.78		2.78	
*p*	0.68		0.75		0.57	

## Data Availability

The data used to support the finding of this study are available from the corresponding author upon request.

## Supplementary Materials

The following supporting information can be downloaded at: https://www.mdpi.com/article/10.3390/molecules27092657/s1, Kalman and CLS-Excel Program.csv; EXP1(Lab Mix).csv.

## Sample Availability

Samples of the Ibuparavic tablets containing PAR, IBU and CAF are available from the authors.
